# Telemedicine application in patients with chronic disease: a systematic review and meta-analysis

**DOI:** 10.1186/s12911-022-01845-2

**Published:** 2022-04-19

**Authors:** Yue Ma, Chongbo Zhao, Yan Zhao, Jiahong Lu, Hong Jiang, Yanpei Cao, Yafang Xu

**Affiliations:** 1 Department of Nursing, Huashan Hospital, Fudan University, 12 Middle Urumqi Road, Shanghai, China; 2 Department of Neurology, Huashan Hospital, Fudan University, Shanghai, China; 3 School of Nursing, Fudan University, Shanghai, China

**Keywords:** Telemedicine, Diabetes, Hypertension, Rheumatoid arthritis, Systematic review, Meta-analysis

## Abstract

**Background:**

Telemedicine has been widely used for long-term care and self-management in patients with chronic disease, but there is no consensus regarding the effect of telemedicine on chronic disease management. The aim of this study is to review and analyse the effect of telemedicine on the management of chronic diseases such as hypertension, diabetes, and rheumatoid arthritis using a systematic review and meta-analysis.

**Methods:**

We performed a comprehensive literature search of the Web of Science, PubMed, MEDLINE, EMBASE, CNKI (Chinese database), VIP (Chinese database), WanFang (Chinese database), and SinoMed (Chinese database) databases from their inception until December 31, 2021. The retrieved literature was screened and assessed independently by two authors. We used the risk-of-bias assessment tool recommended by the Cochrane Handbook for Systematic Reviews of Interventions 5.0.2 for assessing literature quality and Revman 5.3 software to conduct the meta-analysis.

**Results:**

Fifteen articles were included in this study. The results of the systematic review indicated that telemedicine consultation and telemonitoring are the most commonly used intervention methods. Telemedicine is helpful for improving self-management in patients with rheumatoid arthritis. The results of the meta-analysis showed patients’ index of glycosylated hemoglobin (HbA1c) improved after 12 months of intervention (MD =  − 0.84; 95% CI =  − 1.53, − 0.16; Z = 2.42; *P* = 0.02), and no significant differences in fasting blood glucose (FBG) levels were observed after 6 months of intervention (MD =  − 0.35; 95% CI =  − 0.75,0.06; Z = 1.69; *P* = 0.09). The results also showed that systolic blood pressure (MD =  − 6.71; 95% CI =  − 11.40, − 2.02; Z = 2.81; *P* = 0.005) was reduced after 6 months of intervention.

**Conclusion:**

Telemedicine had a positive effect on the management of diabetes, hypertension, and rheumatoid arthritis, especially when telemedicine consultation and telemonitoring method were used. When telemedicine was used as a disease management tool for patients with diabetes, the optimal intervention time is 12 months. Telemedicine improved the systolic blood pressure in hypertensive patients while also reducing negative emotions and enhancing medication adherence in rheumatoid arthritis patients.

## Introduction

During the global COVID-19 pandemic, telemedicine provides a measure for safe social distancing, especially for the long-term care and self-management of patients with chronic disease [1, 2]. Telemedicine is a feasible, credible technology [3], and it has advantages in chronic disease management, such as avoiding the risk of cross-infection between patients. In addition, telemedicine is not limited by time and space requirements. Telemedicine establishes a bridge between healthcare workers and patients for communication and providing medical information [4].

**Fig. 1:**
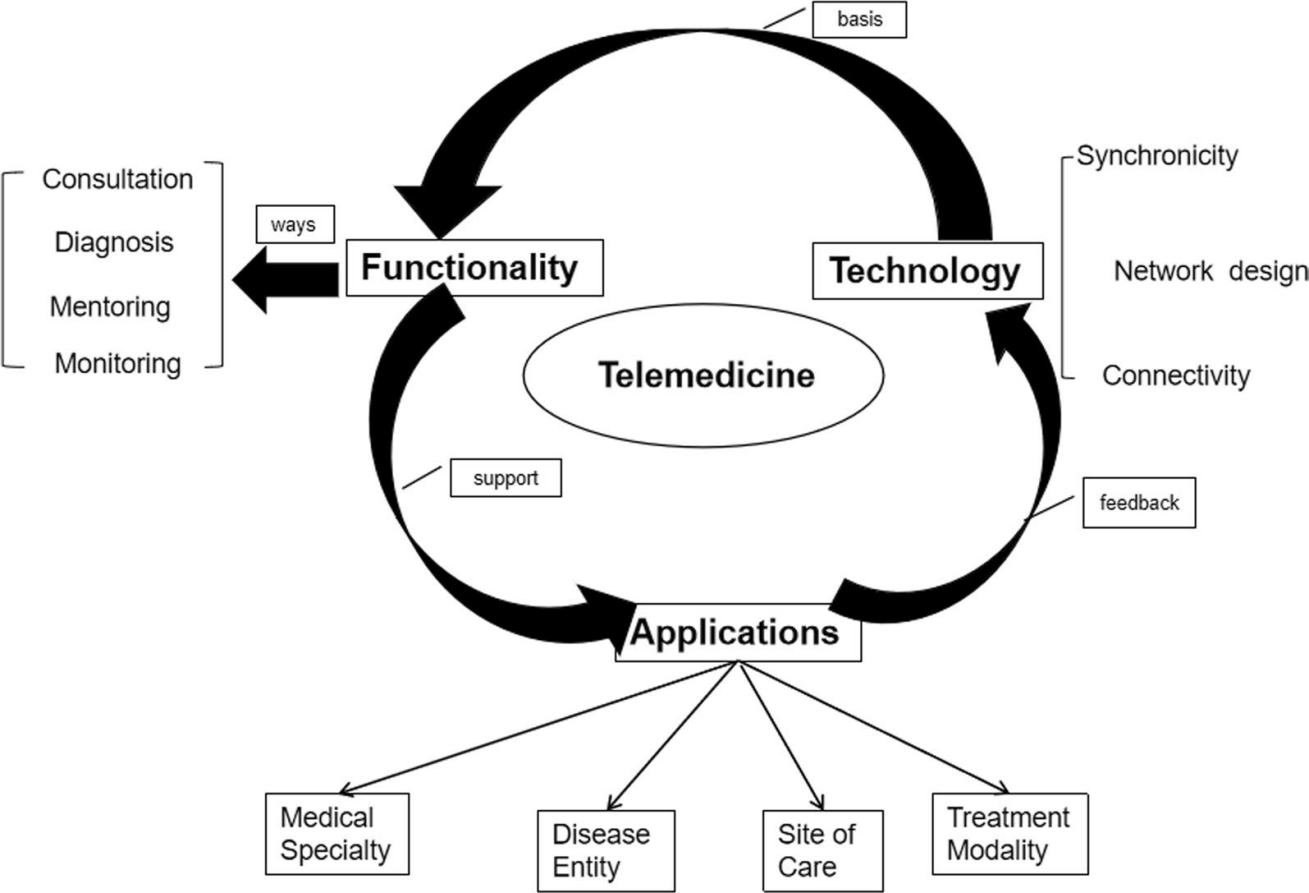
Telemedicine model diagram

Telemedicine is defined by the American Telemedicine Association (ATA) as an electronic tool to communicate medical information and connect the patients and providers in distant locations [5, 6]. Telemedicine can be composed of the following three segments: technology, functionality, and applications [7, 8]. Technology comprises three elements for transmission and exchange: synchronicity, network design, and connectivity. Synchronicity is incorporated with timing and technology, and it is achieved through video conferencing, telemetry, remote sensing, and other modes of interactive health communication. The functions of network design are to post and share information. Wired and wireless connectivity are always used. The functionality of telemedicine consists of several methods, such as consultation, diagnosis, mentoring, and monitoring. All methods aim to allow the transmission of medical information and realize communication between patients and medical workers. But they still have different, telemedicine consultation is defined as patients consulting the specialist via video or chat which is more interactive and human-centered and can be customized to the individual needs of patients [9, 10], telediagnosis is one of the most developed components of telemedicine[11], it makes use of technology to exchange images and data for making a diagnosis of disease[12] and often does not require direct clinical examinations[13], tele-mentoring is used in medical education to disseminate the knowledge and technology, such as fall prevention and infection control [14, 15], telemonitoring involves remote monitoring using mobile devices or computers to monitor the patients’ vital signs or indicators of disease and is usually used by care providers in different locations[16]. Applications are used as platforms that allow for patients and providers to communicate [7] (Fig. 1).

Chronic disease is defined as a disease that has one or more of the following characteristics: it is permanent, leaves residual disability, is caused by a nonreversible pathological alteration, requires special training of the patient for rehabilitation, or the patient may be expected to require a long period of supervision, observation, or care [17]. Chronic diseases seriously affect people's lives due to the following: a decline in the quality of life, impaired mobility, negative emotion, and increase in the economic burden, and a higher mortality rate [18]. In 2012, half of all adults in the United States had at least one chronic disease, and at least one in four had two or more [19]. According to the 2015 World Health Organization (WHO) report, chronic diseases account for 38 million deaths per year [20–22] and have become a public health issue [23]. Hypertension, diabetes, and rheumatoid arthritis are common chronic diseases [6, 24–26]. At least one billion people currently have hypertension worldwide, and it is also predicted that 1.56 billion people will have hypertension by 2025 [24]. According to epidemiological survey results, approximately 180 million people worldwide have diabetes, and this number may double by 2030 [25]. The incidence of rheumatoid arthritis in adults worldwide is 0.5%, and it has become one of the top 10 chronic diseases in China. In addition, the incidence of rheumatoid arthritis in adults ranges from 0.5% to 1.0% in the United States [6, 26]. Hence, more attention should be paid to the aforementioned diseases.

A growing number of studies have focused on telemedicine for chronic disease management. However, there is no consensus on the effect of telemedicine on chronic disease management. Therefore, the aim of this study is to review and analyse the effect of telemedicine on the management of hypertension, diabetes, and rheumatoid arthritis using a systematic review and meta-analysis.

## Methods

### Selection of studies

The inclusion criteria regarding the literature were as follows: (2) the study was a randomized-controlled trial (RCT); (2) telemedicine was the intervention in the study; (3) the disease type must be chronic; and (4) the articles were published in English and Chinese.

### Search strategy

The literature search was performed using the Cochrane, CINAHL, EBSCO, Medline, PubMed, EMBASE, Web of Science, JBI, NICE, SinoMed (Chinese database), CNKI (Chinese database), VIP (Chinese database), and WanFang (Chinese database) databases. The article search period interval for each database was from the inception of the database to December 31, 2021.

The English search terms used for literature retrieval included the following: ("Telemedicine" OR "Remote Consultation" OR " telehealth" OR "telemonitoring" OR "Web-based" OR "mobile monitoring" OR "mobile health" OR" mentoring "OR" internet-based" OR "diagnosis "OR"promoting monitoring "OR" mHealth" OR "telecare") AND ("chronic disease" OR "hypertension" OR "rheumatoid arthritis" OR "diabetes” OR “diabetes mellitus") AND (“disease management” OR “management”). Chinese search terms were also used to conduct literature searches of the Chinese databases.

### Data extraction

The literature was reviewed by two authors, and a third reviewer was consulted when there was uncertainty regarding eligibility. The two authors screened the literature independently according to the title, keywords, abstract, and full-text reading. The data extracted related to the study characteristics, participants, methods of telemedicine, conclusions, and relevant outcomes.

### Risk of bias assessment

The quality of the studies was evaluated using the risk-of-bias assessment tool (RCT recommendation by the Cochrane Handbook for Systematic Reviews of Interventions 5.0.2)[27]. The assessment was independently assessed by two authors. If they had inconsistent opinions after the evaluation, the third author was invited to reassess the quality of the literature.

### Data synthesis

The meta-analysis was performed using Revman 5.3 software. We conducted a meta-analysis of three or more articles that reported the same outcome indicators. We calculated a 95% confidence interval (CI), and the meta-analysis test level was set at *P* = 0.05. I^2^ statistics were used to assess the heterogeneity of effect size, and the I^2^ statistic was used to check for inconsistencies between the studies (I^2^ = 0%–100%; greater than 50% was considered significant statistical heterogeneity). Forest plots were used to visually assess the 95% CIs and *P* values across studies.

## Results

### Results of the literature retrieval

**Fig. 2:**
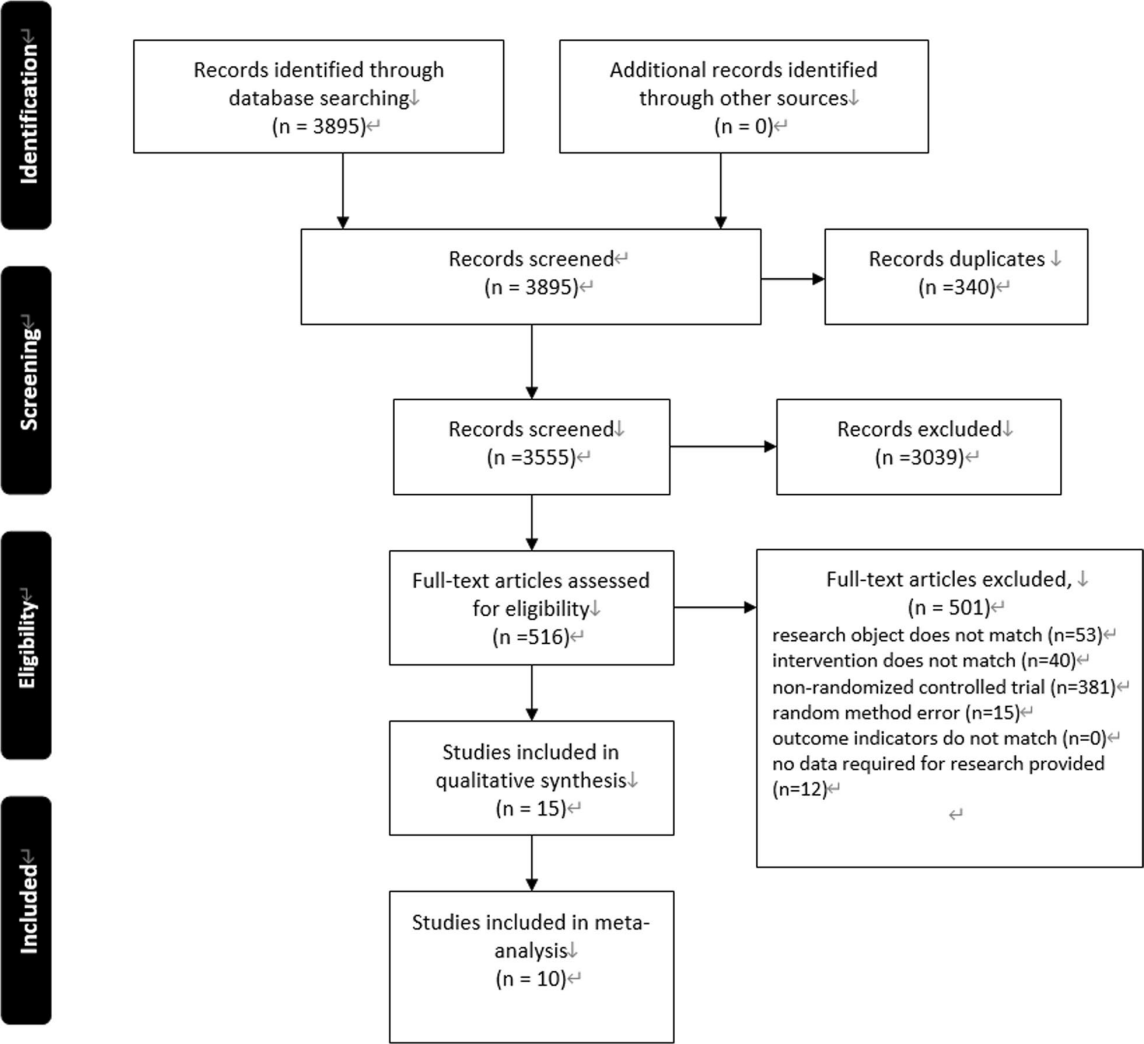
Literature screening flow chart

Figure 2 shows a flowchart of the literature retrieval process according to the Preferred Reporting Items for Systematic Reviews and Meta-Analyses (PRISMA) statement. The search recovered 3895 articles. We excluded 340 duplicates, and 3039 articles were removed after the title and abstract were assessed. The full texts of 516 articles were read, and articles were excluded if research object did not match, the intervention did not match, the study was not an randomized controlled trial, a random method error was present, the outcome indicators did not match, or if no data required for the study were provided. A total of 15 articles were included in the study.

### Risk of bias assessment

**Fig. 3:**
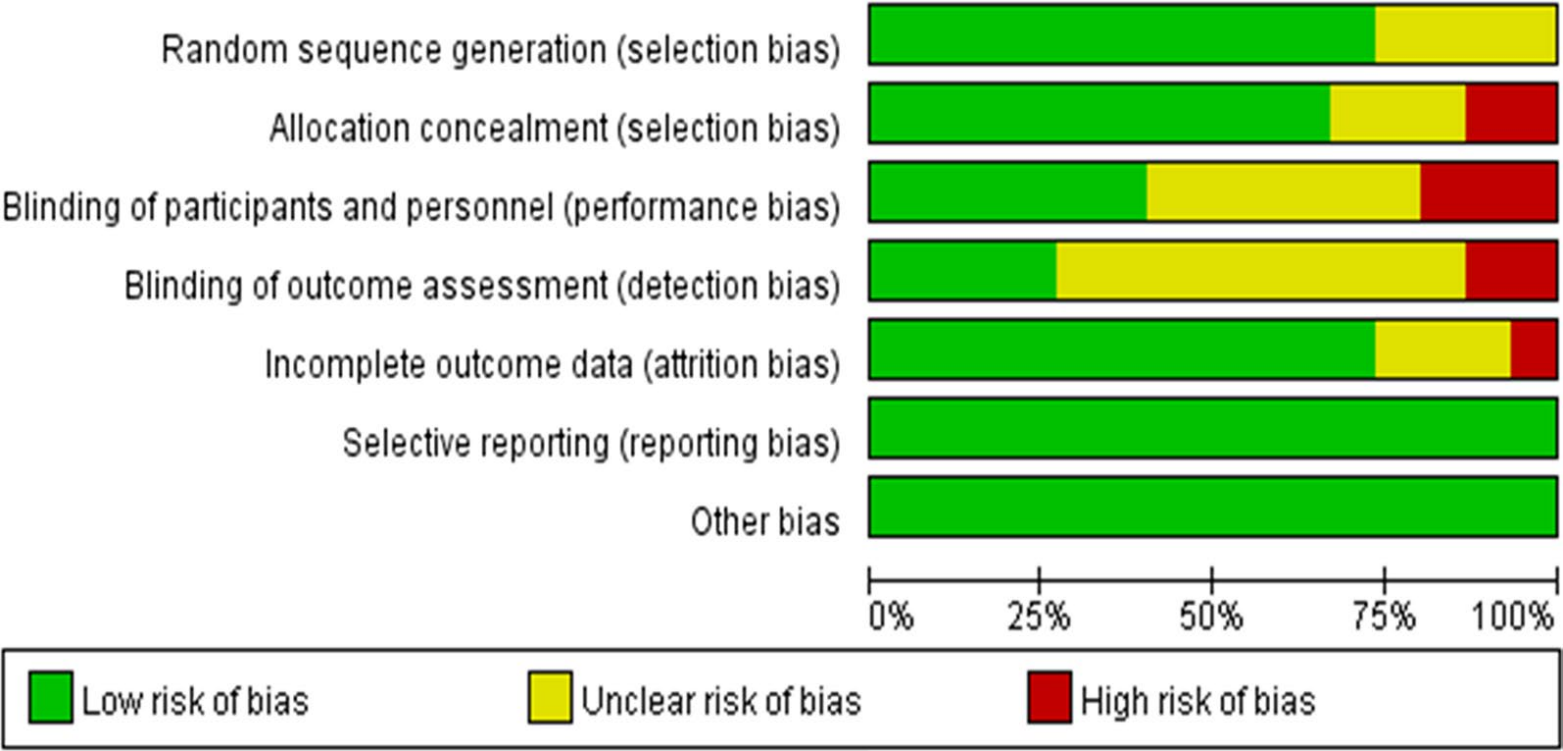
Chart of quality risk assessment of related literature

The characteristics of the studies are shown in Table 1. Figure 3 shows the results of the quality risk assessment. Eleven studies that achieved the generation of a standard random sequence and proper allocation concealment [28–38], and only two studies did not use the allocation concealment method [37, 39]. Four of them used blinded methods for the participants, investigators, and outcome measurers [28, 33, 36, 40, 41], and two studies clearly mentioned that the participants, investigators, and outcome measurers were not blinded due to the intervention methods used [31, 35, 37]. Most of the literature reported the missing data and the reasons for this. All the literature included in this study was considered high quality after the bias assessment.

**Table 1:** Characteristics of included studies

Author, year	Participants with disease	Telemedicine inventions	Intervention providers	Outcome index	Experiment duration	Conclusion
Han Yun et al. [30]	(Diabetes) (1) Experiment sample size:46 (2) Control sample size:45 Age:25–75	Telemedicine inventions: web-based consultation	Endocrinologist; nurse; nutritionist; exercise therapist; doctor	HbA1c FBG PBG	3/6/12 months	FBG was reduced
Kun et al. [40]	(Diabetes) (1) Experiment sample size: 97 Age: 52.6士9.1 (2) Control sample size:89 Age: 54.7土10.3	Telemedicine inventions: Telemonitoring	Nurse Doctor	HbA1C FBG HDL-C LDL-C	3/6 months	FBG, HbA1c were improved
Lee et al. [28]	(Diabetes) (1) Experiment sample size:120 Age: 56.1 ± 9.2 (2) Control sample size:120 Age: 70.9 ± 6.8	Telemedicine inventions: Telemonitoring	Doctor Researcher Clinician	HbA1C FBG BP LDL Total cholesterol	1/3/6/12 months	SBP and total cholesterol were statistically significant
Shea et al. [38]	(Diabetes) (1) Experiment sample size:700 Age: 70.8 ± 6.5 (2) Control sample size:717 Age: 70.9 ± 6.8	Telemedicine inventions: Remote consultation	Nurse case manages; physician	HbA1c LDL BP	12 months	HbA1c, BP and LDL were improved
Shea et al. [39]	(Diabetes) (1) Experiment sample size:729 Age: 70.8 ± 6.5 (2) Control sample size:716 Age: 70.9 ± 6.8	Telemedicine inventions: remote consultation	Nurse case manages; physician	HbA1c LDL BP	12/24/36/48/60 months	HbA1c, BP and LDL levels were improved
Sood et al. [31]	(Diabetes) (1) Experiment sample size:199 Age: 61.6 ± 9.4 (2)Control sample size:83 Age: 61.1 ± 10.0	Telemedicine inventions: Remote consultation	A team of a diabetes specialist (endocrinologist) and a nurse practitioner	HbA1c BP LDL HDL	18 months	HbA1c was no statistically significant
Fountoulakis et al. [26]	(Diabetes) (1) Experiment sample size:70 Age: 55.2 ± 16.1 (2) Control sample size:35 Age: 55.4 ± 19.2	Telemedicine inventions: telemonitoring	Outpatient Department Endocrinologists Doctor	HbA1c BMI Cost	3/6/6 months after discontinuation	Telemonitoring effective in rapidly reducing HbA1c
Lu et al. [35]	(Diabetes) (1) Experiment sample size:60 Age:56.75 ± 12.05 (2) Control sample size:59 Age: 53.17 ± 11.44	Telemedicine inventions: Internet-based consultation	pharmacist	HbA1c FBG edication	6 months	HbA1c and FBG were no statistically significant
Yuli Hu et al. [34]	(Diabetes) (1) Experiment sample size:72 Age:50.04 ± 5.76 (2) Control sample size:70 Age:52.21 ± 8.38	Telemedicine inventions: Internet-based consultation	Doctors Nurses	HbA1c Urinary albumin to creatinine ratio Carotid plaque		The telemedicine system reduced rate of hypoglycemia and indexes of HbA1c
Jia et al. [32]	(Hypertension) (1) Experiment sample size:81 Age: 70.2 ± 6.5 (2) Control sample size:81 Age: 69.2 ± 6.7	Telemedicine inventions: Internet-based consultation	Nurse Doctor	BP	6 months	BP was improved
Margolis et al. [37]	(Hypertension) (1) Experiment sample size:228 Age: 62.0 ± 11.7 (2) Control sample size:222 Age: 60.2 ± 12.2	Telemedicine inventions: telemonitoring	Pharmacists Doctor	BP	6/12/18/54 months	BP was decreased after 12 months
McManus et al. [33]	(Hypertension) (1)Experiment sample size:234 Age: 66.6 ± 8.8 (2) Control sample size:246 Age: 66.2 ± 8.8	Telemedicine inventions: Telemonitoring	Research team; Family doctor	BP	6/12 months	BP changed significantly after 6 months and 12 months
Richard et al.	(Hypertension) (1) Experiment sample size:269 Age: 65.2 ± 10.3 (2) Control sample size:278 Age: 66.7 ± 10.2	Telemedicine inventions: telemonitoring	Healthcare practitioners	BP	6/12 months	The systolic blood pressure was improved after one year than usual care
Song et al. [29]	(Rheumatoid arthritis) (1) Experiment sample size: 41 (2) Control sample size: 36 Age: 55.26 ± 10.84	Telemedicine inventions: telemonitoring	Nurses	Medication compliance Disease activity	3/6 months	The intervention group had significantly higher medication adherence
Ferwerda et al. [27]	(Rheumatoid arthritis) (1) Experiment sample size: 62 (2) Control sample size: 71 Age: 56.35 ± 10.00	Telemedicine inventions: internet-based consultation	Therapists	Beck depression inventory Negative mood Anxiety Satisfaction	3/6/9/12 months	Patients with psychological distress have reduced

*HbA1c* glycosylated hemoglobin; *FBG* fasting blood glucose; *PBG* postprandial blood glucose; *FPG* fasting plasma glucose; *HDL-C* high-density lipoprotein cholesterol; *LDL-C* low-density lipoprotein cholesterol; *BMI* Body Mass Index; *BP* blood pressure; *LDL* low density lipoprotein; *HbA1c* glycosylated hemoglobin; *HDL* high density lipoprotein; *SBP* systolic blood pressure; *DBP* diastolic blood pressure

### Systematic review of the effects of telemedicine in patients with hypertension, diabetes, and rheumatoid arthritis

#### Telemedicine intervention methods

Telemedicine consultation and telemonitoring are the most commonly used telemedicine intervention methods in patients with diabetes, hypertension and rheumatoid arthritis.

A total of nine articles used telemedicine consultation and telemonitoring as telemedicine intervention methods in patients with diabetes [28, 30, 32, 33, 36, 37, 40–42]. Han et al. studied the effect of telemedicine consultation in the disease management of diabetes, and the study showed that patients’ fast blood glucose (FBG) levels were reduced after a 12-month intervention [32]. However, Sood et al. obtained a different result and found that the HbA1c index did not improve after an intervention of 14.8 months using telemedicine consultation. This result was not significant when compared with the control group [33]. In a study by Feng et al., the FBG and HbA1c indexes were improved using telemonitoring as an intervention [42]. Lee et al. evaluated the effects of telemonitoring with team-based management on patients with diabetes, and they reported an improvement in glucose after 24-week and 52-week interventions [30]. Shea et al. used both telemedicine consultation and telemonitoring as interventions for home unit management and nursing case management in patients with diabetes. The results showed that the patients’ HbA1c indexes improved in the experimental group [40, 41]. Fountoulakis et al. also found that telemonitoring that combined a management and feedback system based on transmitted data helped to improve HbA1c indexes [28]. Lu et al. used telemedicine consultation as an intervention which included pharmacy consultation, medication evaluation, and treatment, overall, they found that telemedicine consultation was an effective method for patients with diabetes[37]. Hu et al. also found the telemonitoring of blood glucose levels could reduce the HbA1c index in patients with diabetes[36].

Four articles described the use of telemedicine consultation and telemonitoring as telemedicine interventions in patients with hypertension [34, 35, 38, 39]. In a study conducted by Li et al., out a total of 162 participants were recruited and the experimental group received telemedicine consultation as the intervention method. They found that the patients’ blood pressure improved after 6 months of intervention [34]. Another study investigated the durability of the intervention effect on blood pressure, and they using home blood pressure telemonitoring in the experimental group. The results showed that a change in blood pressure began after 12 months [39]. McManus et al. also used telemonitoring of home blood pressure measurements for the self-management of hypertension. They found that it assisted in reducing blood pressure after 6 and 12 months [35]. However, Richard et al. used telemonitoring intervention for self-management which can provide detailed feedback. The result showed that compared to typical care, the telemonitoring of blood pressure led to better control of systolic blood pressure after one year [38].

Telemedicine consultation as a telemedicine intervention has also been used for patients with rheumatoid arthritis [29, 31]. A study by Ferwerda et al. evaluated the effect of telemedicine consultation on patients with rheumatoid arthritis. The results showed that the telemedicine consultation reduced patients’ anxiety and depression [29]. Song et al. conducted a study to investigate the impact of telemedicine on drug compliance and disease activity in rheumatoid arthritis patients. They found that telemedicine consultation did not improve patient’s symptoms but it did enhance medication compliance [31].

#### Outcome measures

A total of fifteen articles on the application of telemedicine for disease management were included in the quality evaluation of the literature [28–42]. Six of fifteen articles were related to diabetes, and the outcome indicators included the HbA1c and the FBG indexes. We also compared the effects of telemedicine management after 6 and 12 months of intervention [30, 32, 36, 37, 40, 42]. Four articles described hypertension, and they had the same intervention duratio. Therefore, these ten articles were included in the meta-analysis [30, 32, 34–40, 42]. The remaining five articles were included in a systematic review as follows [28, 29, 31, 33, 41].

Six articles described the use of HbA1c and FBG as the primary outcomes [30, 32, 36, 37, 40, 42]. High FBG is a potential risk factor for small arterial stiffness and it tends to cause diabetic complications [43]. HbA1c, an indirect measure of the mean blood glucose index, reflects the blood glucose level over the previous 2 to 3 months [44]. A meta-analysis of HbA1c was performed according to the 6- and 12-month intervention durations [30, 32, 36, 37, 40, 42].

Another four articles covered disease management for hypertension [34, 35, 38, 39], and telemedicine was the primary intervention method. All of the researchers used systolic blood pressure and diastolic blood pressure as the primary outcomes to reflect blood pressure fluctuations. Hence, we conducted a meta-analysis to test the effect of the intervention.

### Results of the meta-analysis

#### Meta-analysis of the effects of telemedicine in diabetes patients

Six articles used HbA1c and FBG to evaluate the effect of telemedicine on diabetic patients, and we conducted a meta-analysis based on the outcome index and duration of intervention [30, 32, 36, 37, 40, 42].

##### Meta-analysis of HbA1c after 6 months of intervention

**Fig. 4:**
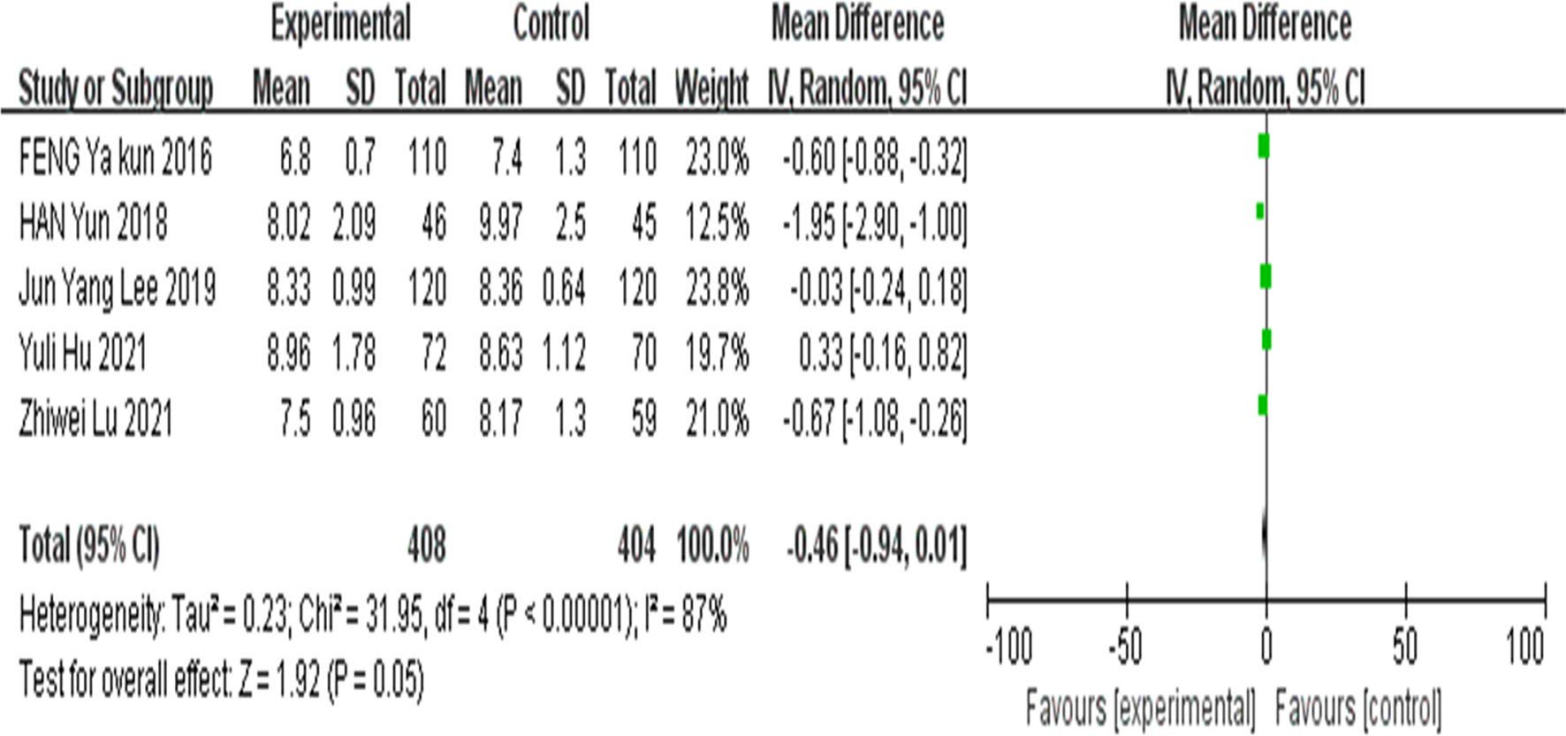
Forest plots of glycosylated hemoglobin after 6 months of intervention between the intervention group and the control group

A total of five articles described HbA1c after 6 months of telemedicine intervention. The results showed that the I^2^ value was 87%, indicating high heterogeneity, thus, we adopted a random-effects model which showed no significant difference in HbA1c indexes between the intervention group and the control group after 6 months of intervention. (MD =  − 0.46; 95% CI =  − 0.94, 0.01; Z = 1.92; *P* = 0.05) (Fig. 4).

##### Meta-analysis of HbA1c after 12 months of intervention

**Fig. 5:**
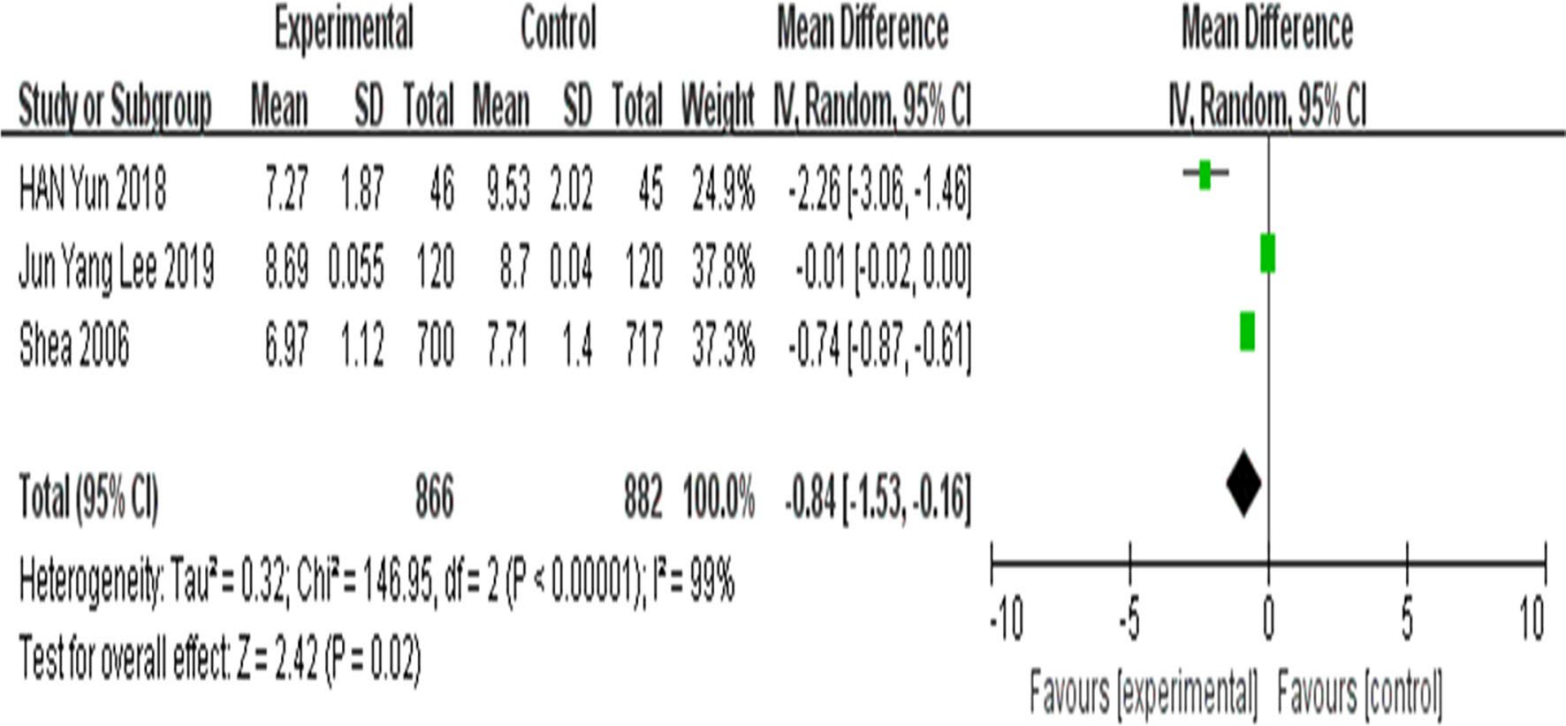
Forest plots of glycosylated hemoglobin after 12 months of intervention between the intervention group and the control group

Because of the high statistical heterogeneity (I^2^ = 99%), the random-effects model was used. The results showed that there were statistically significant differences in HbA1c indexes between the intervention and control groups [30, 32, 40] (MD =  − 0.84; 95% CI =  − 1.53, − 0.16; Z = 2.42; *P* = 0.02) (Fig. 5).

##### Meta-analysis of FBG after 6 months of intervention

**Fig. 6:**
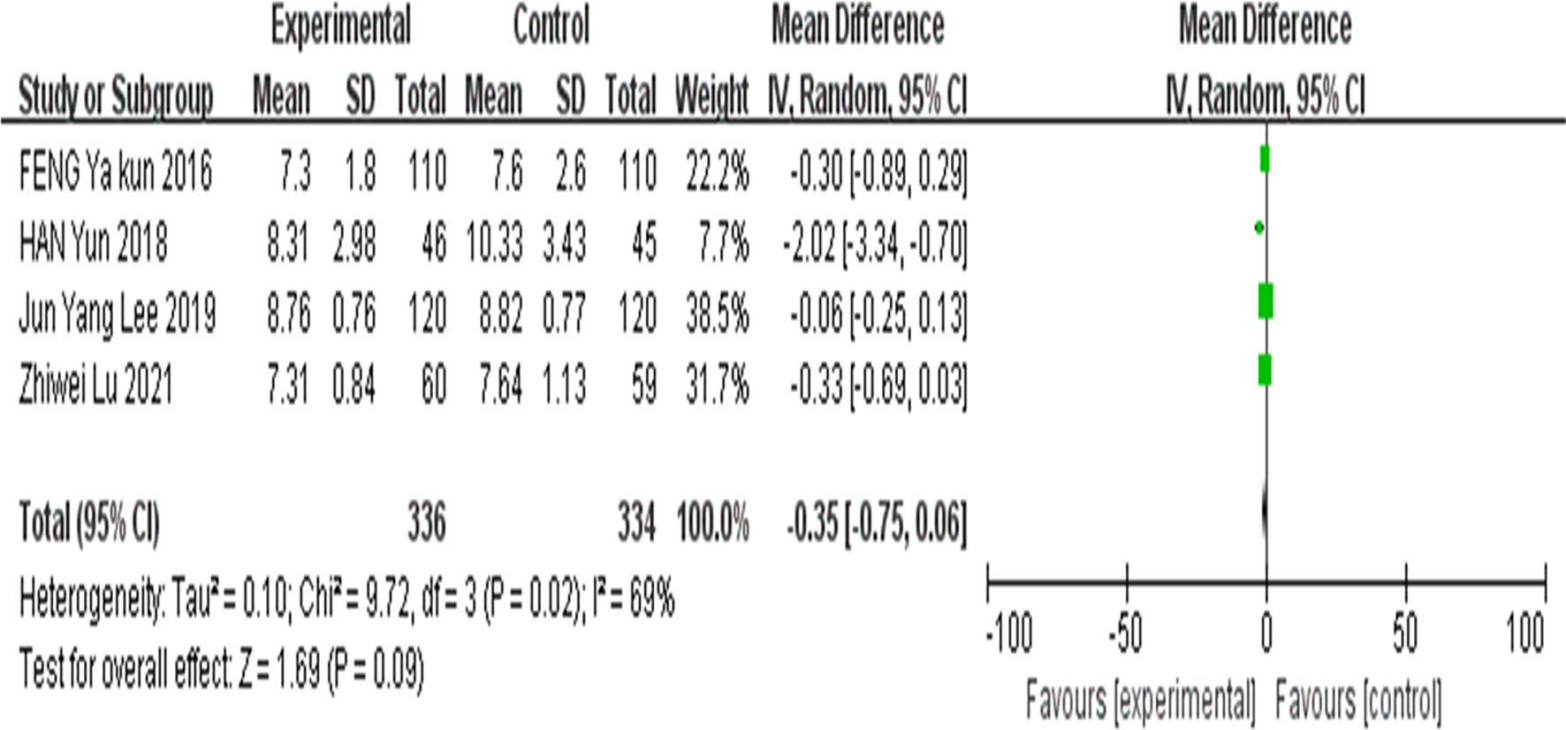
Forest plots of fasting blood glucose after 6 months of intervention between the intervention group and the control group

This set of data was analysed using a random-effects model due to its high statistical heterogeneity (I^2^ = 69%). The results showed that there was no significant difference in FBG levels between the experimental group and the control group (MD =  − 0.35; 95% CI =  − 0.75,0.06; Z = 1.69; *P* = 0.09) (Fig. 6).

#### Meta-analysis of the effect of telemedicine in hypertension patients

In this study, we included four articles that investigated the effect of telemedicine on patients with hypertension. Four reported the index of systolic blood pressure and diastolic blood pressure after 6 months of intervention. We conducted the meta-analysis according to the outcome index and duration of intervention [30, 35, 38, 39].

##### Meta-analysis of the changes in systolic blood pressure

**Fig. 7:**
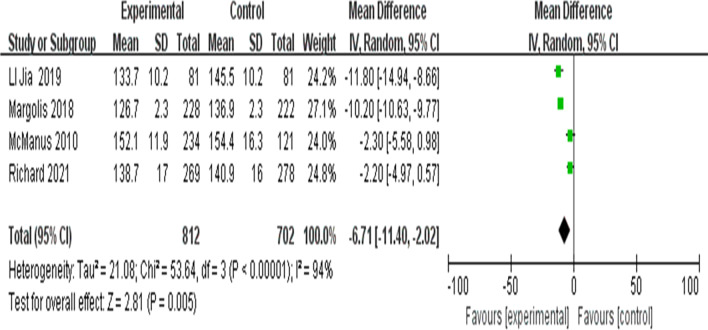
Forest plots of the systolic blood pressure after 6 months of intervention between the intervention group and the control group

The systolic blood pressures in the experimental and the control groups after 6 months of intervention in the included literature were analysed. A random-effects model was adopted since there was high statistical heterogeneity (I^2^ = 94%). The results showed that there were statistically significant differences in systolic blood pressure between the group receiving telemedicine and the control group (MD =  − 6.71; 95% CI =  − 11.40, − 2.02; Z = 2.81; *P* = 0.005) (Fig. 7).

##### Meta-analysis of the changes in the diastolic blood pressure

**Fig. 8:**
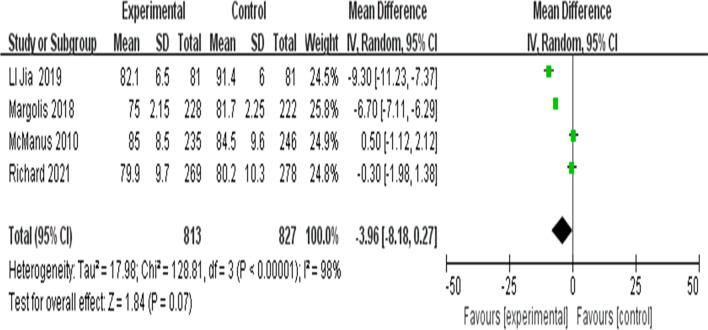
Forest plots of diastolic blood pressure after 6 months of intervention between the intervention group and the control group

Four articles described diastolic blood pressure at 6 months of intervention [34, 35, 38, 39]. A random-effects model was used because there was high statistical heterogeneity (I^2^ = 98%). The results showed that there were no statistically significant differences in diastolic blood pressure between the intervention and control group (MD =  − 3.96; 95% CI =  − 8.18, 0.27; Z = 1.84; *P* = 0.07) (Fig. 8).

## Discussion

### Telemedicine consultation and telemonitoring are the most commonly used methods of telemedicine intervention

Telemedicine can provide long-term care and treatment for people with chronic diseases via an online application or other telecommunication technology, and this is helpful for maintaining a safe social distance. Telemedicine consultation and telemonitoring are common telemedicine methods. Telemedicine consultations meet the current health care needs of patients while demonstrating convenience. Patients are usually satisfied with the service and have the ability to choose when to conduct consultations [10]. Telemonitoring reduces the number of visits and hospital admissions, thus, scarce medical resources can be used effectively [45]. Many studies have confirmed that telemedicine technologies are beneficial as a medical resource, and telemedicine can also help to adjust care plans in a timely manner [46]. Telemedicine has also been considered a cost-effective method for long-term care [47]. However, there still exist large barriers for patients that have difficulty and experience anxiety when using a computer or mobile phone as a tool to receive telemedicine, especially older individuals [48].

### The duration of telemedicine intervention is important for HbA1c index improvement in diabetes patients

HbA1c indexes were significantly improved after 12 months of telemedicine intervention compared with 6 months, and the longer-term intervention had positive effects on the controlling HbA1c indexes. The HbA1c index reflects the average level of blood glucose over a period of time, and this index can change slowly. Hence, a long intervention period may be necessary for improvements to be observed. Thus, the results of the longer intervention period were consistent with other studies that obtained increased HbA1c changes over time [44]. This result was similar to that of Timpel et al., who found that HbA1c began to decrease after up to 12 months of long-term telemedicine intervention [49]. Another study also revealed this phenomenon. A Spanish study of 328 diabetic patients, provided the intervention group with a tele-assistance system that transmitted blood glucose results in real-time and utilized remote consultation. The control group was followed up with regularly at their healthcare centre, notably, one year later, researchers found that the HbA1c indexes were significantly decreased in the intervention group compared with those in the control group [50].

However, the results of this study showed that the change in FBG was not statistically significant after 6 months of telemedicine intervention. Taylor et al. showed that the sensitivity of FBG levels for the diagnosis of diabetes is low, and there are substantial differences between populations[51, 52]. Moreover, FBG needs to be measured after fasting for 8 h or more. Because FBG fluctuates greatly during the day and night, FBG levels measured at the same time point should be compared. Notably, the literature included in this study did not indicate the time that FBG was measured. Therefore, more research is needed to verify the impact of telemedicine intervention on FBG levels.

### Systolic blood pressure was improved after telemedicine intervention for 6 months in hypertension patients

There were significant improvements in systolic blood pressure after 6 months of intervention. Although different durations of interventions were reported in patients with hypertension, most patients experienced a significant drop in blood pressure after 6 months of telemonitoring [53–56]. Telemedicine yielded positive effects on the management of hypertension patients, especially after a 6-month intervention. Thus, telemedicine technology could support doctors and nurses in regard to the close, continuous follow-up of hypertension patients.

### Telemedicine had positive effects on rheumatoid arthritis patients

Rheumatoid arthritis is one of the most prevalent chronic inflammatory diseases. The primary symptoms of rheumatoid arthritis include rheumatoid nodules, pulmonary involvement or vasculitis, and systemic comorbidities [57]. Patients with rheumatoid arthritis have a long disease duration involving joint deterioration and functional disability, eventually leading to unfavourable disease outcomes. Patients with rheumatoid arthritis often experience psychological distress, such as anxiety and depression, and all of these symptoms seriously affect daily life activities [58]. The method for the long-term management of rheumatoid arthritis patients with telemedicine has developed gradually. Telemedicine as the primary management method among rheumatoid arthritis patients could improve negative emotions and promote medication adherence. More studies should be performed in the future to confirm the effect of telemedicine on the disease management of rheumatoid arthritis.

## Conclusion

Here, we conducted a systematic review and analyse to review and analysis the effect of telemedicine on patients with hypertension, diabetes, and rheumatoid arthritis. The results demonstrated that telemedicine consultation and telemonitoring are the primary methods used for telemedicine intervention. This study also indicated that telemedicine had a positive effect on the management of hypertension, diabetes, and rheumatoid arthritis, and that telemedicine was effective in regard to the systolic blood pressure of hypertensive patients after intervention for 6 months. Long-term telemedicine interventions showed a significant effect on HbA1c index management in diabetic patients. Thus, telemedicine has the ability to substantially improve the quality of disease management [59].

The results of this study indicate that telemedicine should be recommended as a useful tool for disease management in patients with chronic diseases such as hypertension and diabetes. Telemedicine could also improve negative emotions and promote medication adherence in rheumatoid arthritis patients. The results indicate that 12 months of telemedicine intervention should be considered for diabetic patients.

## Data Availability

All data generated or analysed during this study are included in this published article [and its supplementary information files].
